# Supplementary material for: Rapid bacteriophage quantification with a particle size analyzer combined with polarization intensity differential scattering (PIDS) detector
Source: PLoS One. 2026 Feb 19;21(2):e0343131. doi: 10.1371/journal.pone.0343131 (PMC12919812; doi:10.1371/journal.pone.0343131)
Supplement: Attachment — Submitted filename: PONE-D-25-61226_reviewer.docx [file pone.0343131.s003.docx]

**Rapid bacteriophage quantification with a particle size analyzer combined with Polarization Intensity Differential Scattering (PIDS) detector**

**1. Abstract (Lines 22–34):**

-The abstract lacks quantitative results; it does not provide numerical values, particle size ranges, or statistical indicators. Additionally, the final sentence fails to clearly state the main conclusion or its implications.

-Inconsistent use of verb tenses was observed. The entire abstract should be rewritten in the past simple tense. For example, in line 23, "has expanded" (present perfect) and in line 33, "We demonstrate" (present simple) should both be converted to the past simple tense.

- The limit of detection is not mentioned in the abstract.

**2. Introduction and Literature Review (Lines 42–86):**

-The literature review is fragmented and lacks logical coherence. Several references are outdated and should be replaced with more recent studies published after 2019. For example, references cited on lines 392 (2000) and 395 (2009) are outdated. It is recommended to include more recent systematic reviews published after 2020.

-The explanation of fluorescent microscopy methods in lines 64–75 is somewhat lengthy and could be more concise.

- The scientific knowledge gap is not clearly defined. The novelty and specific contribution of the study remain unclear. Although lines 87–96 mention the limitations of existing methods, they do not explicitly state the specific problem that this new method addresses, which others cannot solve.

- It should be explicitly stated in lines 96–107 that this is the first application of PSA combined with PIDS for phage counting.

**3. Methods, Sample Preparation Procedures, and Results (Lines 134–152):**

- The sample preparation procedures are inadequately described. Key details such as initial concentrations, incubation times, and temperatures are missing. Although lines 136–139 mention an incubation time of 3–4 hours at 37°C, the initial concentrations of bacteria or phages are not provided.

- Line 141: The text mentions a µm syringe filter, but it does not specify the brand or the material of the filter.

- Critical details regarding the Amicon Ultra protocol, including membrane cutoff, centrifugation speed, duration, and washing steps, are missing, severely limiting reproducibility. While the cutoff (10 kDa MWCO) and speed (4000 rpm) are specified, the exact centrifugation time—stated only as volume is reduced to 200–500 µL vague. Additionally, washing or equilibration steps are not described. The centrifugation temperature should also be provided.

- The exact volumes of the bacterial suspension and phage dilution are unclear, and the order of mixing is not specified. Line 171 mentions the bacterial volume (250 µL) and phage volume (10 or 100 µL), but the bacterial concentration (OD600) is not reported. Although the mixing order is implied, it could be stated more explicitly.

- Line 175: “Plates were allowed to solidify completely,” but the solidification time is not specified.

- No information is provided regarding the number of replicates or the use of positive and negative controls. Line 154 mentions “in triplicate three separate times,” but negative controls (e.g., blank buffer) and positive controls (e.g., standard particles) are not clearly described.

- The Particle size distribution results are presented without adequate textual interpretation or clear connection to the main research objective. Although lines 263–267 report peak ranges, the interpretation of multi-peak phenomena (e.g., aggregation) is not thoroughly addressed in the Results section and is instead deferred to the Discussion.

**4. Discussion Section (Lines 325–365):**

- The discussion primarily reiterates the results instead of offering interpretation. Comparisons with previous studies are limited. Although lines 334–353 explain the reasons for size distributions, direct comparisons with other counting methods (e.g., flow cytometry) are minimal.

- The Practical limitations of the method, such as the cost of the instruments, should be emphasized more in the Discussion section.

- **Grammatical and stylistic errors (throughout):**

- Numerous grammatical errors, overly long sentences, and inconsistent tense usage are present. A major revision of the English language is required.

Line 64: “Sybr” should be “SYBR.”

Line 148: The term “CsCl” is used without prior explanation; it should be introduced as cesium chloride (CsCl) when first mentioned to ensure clarity.

Line 306: “We needed to understand” should be changed to “We needed to understand” in the past simple tense.

- Long sentences, such as those in lines 73–75, could be broken up for better readability.

- In the References section (lines 390–460), there are formatting inconsistencies, such as irregular capitalization and punctuation.
