# Supplementary material for: Rapid bacteriophage quantification with a particle size analyzer combined with polarization intensity differential scattering (PIDS) detector
Source: PLoS One. 2026 Feb 19;21(2):e0343131. doi: 10.1371/journal.pone.0343131 (PMC12919812; doi:10.1371/journal.pone.0343131)
Supplement: Attachment — Submitted filename: Response to Reviewers.docx [file pone.0343131.s005.docx]

The authors would like to thank the reviewers for taking the time and effort necessary to critique this article and provide suggestions for its betterment. Please find our responses to comments and suggestions below.

Reviewer 1:

1. The abstract lacks quantitative results; it does not provide numerical values, particle size ranges, or statistical indicators. Additionally, the final sentence fails to clearly state the main conclusion or its implications.

*We have revised the abstract to include the range of phage sizes tested and statistics for PSA measurements. We have also stated our main conclusion and implications more clearly. See lines 20-27*

2. Inconsistent use of verb tenses was observed. The entire abstract should be rewritten in the past simple tense. For example, in line 23, "has expanded" (present perfect) and in line 33, "We demonstrate" (present simple) should both be converted to the past simple tense.

*Verb tenses are now consistent.*

3. The limit of detection is not mentioned in the abstract.

*The limit of detection has been added to the abstract. See lines 24-25*

4. The literature review is fragmented and lacks logical coherence. Several references are outdated and should be replaced with more recent studies published after 2019. For example, references cited on lines 392 (2000) and 395 (2009) are outdated. It is recommended to include more recent systematic reviews published after 2020.

*There are limited systematic reviews on phage quantitation methods. We have added more recent references relating to flow cytometry detection of phages.*

5. The explanation of fluorescent microscopy methods in lines 64–75 is somewhat lengthy and could be more concise.

*We have rewritten this section to be more succinct. See lines 53-57.*

6. The scientific knowledge gap is not clearly defined. The novelty and specific contribution of the study remain unclear. Although lines 87–96 mention the limitations of existing methods, they do not explicitly state the specific problem that this new method addresses, which others cannot solve.

*We now state that this method uses a rapid, label-free method that does not require knowledge of the bacterial host (Lines 92-95).*

7. It should be explicitly stated in lines 96–107 that this is the first application of PSA combined with PIDS for phage counting.

*We have done this (Line 92)*

8. The sample preparation procedures are inadequately described. Key details such as initial concentrations, incubation times, and temperatures are missing. Although lines 136–139 mention an incubation time of 3–4 hours at 37°C, the initial concentrations of bacteria or phages are not provided.

*More information has been added. See throughout methods section.*

9. Line 141: The text mentions a µm syringe filter, but it does not specify the brand or the material of the filter.

*The filter material has been added (PTFE). Several brands have been used with all being equally effective, as such no one brand is listed.*

10. Critical details regarding the Amicon Ultra protocol, including membrane cutoff, centrifugation speed, duration, and washing steps, are missing, severely limiting reproducibility. While the cutoff (10 kDa MWCO) and speed (4000 rpm) are specified, the exact centrifugation time—stated only as volume is reduced to 200–500 µL vague. Additionally, washing or equilibration steps are not described. The centrifugation temperature should also be provided.

*Additional information has been added to the protocol where necessary. Speed, temperature, duration, and cutoff have all been included. The time of centrifugation has been put in but is a suggested starting point as it can be insufficient for generating the final 200-500ul volume. Getting to that final volume is the more important point. Washing and equilibration steps are not performed, just centrifugation to concentration. See lines 119-123.*

11. The exact volumes of the bacterial suspension and phage dilution are unclear, and the order of mixing is not specified. Line 171 mentions the bacterial volume (250 µL) and phage volume (10 or 100 µL), but the bacterial concentration (OD600) is not reported. Although the mixing order is implied, it could be stated more explicitly.

*Text states using overnight cultures of bacteria with added information of how long growth was allowed. OD600 is variable in overnight cultures but as long as there is turbid growth, it is sufficient for this protocol to work. Aiming for a specific OD600 is not necessary if using an overnight culture. Mixing order has been clarified further. See lines 143-157.*

12. Line 175: “Plates were allowed to solidify completely,” but the solidification time is not specified.

*No solidification time is listed as it varies by environmental conditions. The important thing is to have it completely solidified prior to inversion for incubation overnight.*

13. No information is provided regarding the number of replicates or the use of positive and negative controls. Line 154 mentions “in triplicate three separate times,” but negative controls (e.g., blank buffer) and positive controls (e.g., standard particles) are not clearly described.

*Description of controls has been added where necessary. In methods, use of bacteria alone is described as the negative control for plaquing assays (Line 161). Use of SM Buffer alone has been described as the negative control for PSA experiments (Lines 249-251).*

14. The Particle size distribution results are presented without adequate textual interpretation or clear connection to the main research objective. Although lines 263–267 report peak ranges, the interpretation of multi-peak phenomena (e.g., aggregation) is not thoroughly addressed in the Results section and is instead deferred to the Discussion.

*We have added the observation and associated reference to explain the peak distribution to the results section (lines 270-272) and have left the more detailed description in the discussion section.*

15. The discussion primarily reiterates the results instead of offering interpretation. Comparisons with previous studies are limited. Although lines 334–353 explain the reasons for size distributions, direct comparisons with other counting methods (e.g., flow cytometry) are minimal.

*We have expanded in the discussion to include further discussion on interpretation, comparison with other counting methods, and instrumentation cost.*

16. The Practical limitations of the method, such as the cost of the instruments, should be emphasized more in the Discussion section.

*We have included the instrument cost relative to instrumentation for other methods. See line 322.*

17. Numerous grammatical errors, overly long sentences, and inconsistent tense usage are present. A major revision of the English language is required.

*We have reviewed with focus on clarification of the language and matching verb tense.*

18. Line 64: “Sybr” should be “SYBR.”

*This has been changed. See line 54.*

19. Line 148: The term “CsCl” is used without prior explanation; it should be introduced as cesium chloride (CsCl) when first mentioned to ensure clarity.

*CsCl has been defined at its first use in line 126*

20. Line 306: “We needed to understand” should be changed to “We needed to understand” in the past simple tense.

*We removed this*

21. Long sentences, such as those in lines 73–75, could be broken up for better readability.

*We have reviewed and changed sentence structure to be succinct*

22. In the References section (lines 390–460), there are formatting inconsistencies, such as irregular capitalization and punctuation.

*References were input using EndNote Vancouver style of citation*

Reviewer #2: This study introduces a novel application of a particle size analyzer with PIDS technology (PhageFOTO) for rapid quantification of bacteriophages. The core idea is innovative and addresses a genuine need for faster, label-free phage enumeration. The method is validated against the plaque assay using three distinct phages, demonstrating promising accuracy. However, the manuscript in its current form requires revision to improve clarity, methodological rigor, and scholarly presentation before it is suitable for publication.

*We thank this reviewer for their input. We have edited this article substantially to increase clarity, better demonstrate our methodological rigor, and improve presentation of our results and discussion.*
